# Associations of inflammatory polyarthritis with clinical and radiological findings of temporomandibular disorders

**DOI:** 10.1080/00016357.2023.2267118

**Published:** 2024-03-22

**Authors:** Sari Oksanen, Kirsi Sipilä, Markku Heliövaara, Anna Liisa Suominen, Sisko Huumonen

**Affiliations:** a Institute of Dentistry, University of Eastern Finland, Kuopio, Finland; b Research Unit of Oral Health Sciences, University of Oulu, Oulu, Finland; c Medical Research Center, Oulu University Hospital and University of Oulu, Oulu, Finland; d Finnish National Institute for Health and Welfare, Helsinki, Finland; e Institute of Dentistry, Kuopio University Hospital, Kuopio, Finland; f Department of Public Health and Welfare, National Institute for Health and Welfare, Helsinki, Finland

**Keywords:** Temporomandibular disorders, polyarthritis, arthritis, rheumatoid, biomarkers

## Abstract

**Objective:**

To evaluate the association of different forms of inflammatory polyarthritis with clinical signs of temporomandibular disorders (TMD) and radiological findings in temporomandibular joint (TMJ), based on a nationwide health survey in Finland. The aim was also to assess the presence of clinical and radiological TMD findings in subjects with increased risk for developing rheumatoid arthritis (RA).

**Material and methods:**

A nationally representative sample included 6331 Finnish adults who participated in the Health 2000 Survey (BRIF8901). Subjects were examined for signs of TMD, findings in panoramic radiograph of TMJ, musculoskeletal health and serology (rheumatoid factor, RF, and anti-cyclic citrullinated peptide, aCCP).

**Results:**

Sixty-four percent of seronegative RA and 60% of seropositive RA subjects had at least one sign of TMD. While adjusting for confounding factors (gender, age, dentures and smoking history), RA was significantly associated with crepitation and abnormal radiological findings in TMJ. Seronegative RA was also associated with restricted mouth opening. Systemic autoimmunity associated with RA (“at risk of RA”) was not associated with clinical or radiological TMD findings.

**Conclusions:**

Clinical and radiological findings of TMD are more prevalent among subjects with inflammatory polyarthritis than among the population in general in the Finnish adult population.

## Introduction

Temporomandibular disorders (TMD) include pain and dys-functions related to the temporomandibular joints (TMJs), masticatory muscles and other adjacent structures of the masticatory system. TMD has multifactorial etiology, including biopsychosocial concepts. Typical symptoms of TMD include pain in facial/jaw area, or TMJs, headache, difficulties in jaw opening and TMJ noises. Common signs of TMD are pain in the masticatory muscles or TMJs, joint sounds such as clicking or crepitation and limited movements of the jaw. Diagnostics of TMD is based on symptom report and clinical examination. Radiographic imaging of TMJs is used as a supplementary method for TMD diagnostics [1]. Based on panoramic radiographs, radiological findings of TMJ may include those related to arthrosis/arthritis and they can reveal osseous changes such as osteophytes and erosion, flattening or sclerosis of the articular surfaces [2].

TMJ is a synovial joint and it can therefore be subject to the symptoms of inflammatory polyarthritis, such as rheumatoid arthritis (RA), causing signs and symptoms of TMD [3]. RA is an inflammatory autoimmune disease. Therefore, its diagnosis also includes positive levels of two serum autoanti-bodies (rheumatoid factor RF and anti-cyclic citrullinated peptide aCCP) in addition to the clinical signs and symptoms [4]. These autoantibodies can be present in the serum up to 10 years before the onset of the clinical manifestations of RA [4,5]. Yet, some patients have clinical manifestations of RA but are persistently negative for these autoantibodies, and these cases are referred as seronegative RA.

In general, TMD are relatively common within the population. A large population-based study showed that the incidence for TMD was nearly 4% per year [6]. The prevalence of clinical signs is commonly higher than TMD related symptoms [7–9]. For example, over third of the Finnish population showed clinical signs of TMD whereas less than one-tenth reported pain symptoms related to the TMD [8]. Based on a recent review, TMD are also common among children and adolescents, the prevalence levels varying from 20 to 60% [10]. Several studies indicate that both signs and symptoms are more frequent in women than in men [7,8,11]. Other factors such as poor general health and psychosocial factors can also increase the risk of TMD [6,12]. Amongst patients with RA, studies have shown frequency of TMD related signs and symptoms from *ca.* 3 to 88%, frequencies over 50% being the most reported [13–20]. Large range in reported frequencies can be partly explained by differences in TMJ assessment and diagnostic criteria. Reported radiological signs in TMJ also range widely, from 13 to 75% in RA patients [13–15].

TMD related to rheumatic diseases can lead to pain, impairments in swallowing and masticatory function [21], occlusal changes [3] as well as reduced quality of life [22,23]. Therefore, its recognition and early treatment is crucial [21,24]. Population-level studies of TMD related to rheumatic diseases are rare [19,25], although they can give comprehensive insight into the frequency of TMD in subjects with rheumatic diseases in relation to the population in general. There exists also only very limited knowledge on the frequency of TMD in subjects having an increased risk of developing RA as indicated by positive levels of related serum autoantibodies [20,26]. Therefore, research on this specific topic is needed. The aims of this study were to evaluate the association of different forms of inflammatory polyarthritis with clinical TMD signs and radiological findings in TMJ, based on panoramic radiography, in a nationwide health survey in Finland. The aim was also to assess the presence of clinical TMD signs and radiological TMJ findings in subjects with increased risk for developing RA.

## Material and methods

### Data collection

A comprehensive multidisciplinary health survey, Health 2000 Study, was carried out in Finland in years 2000–2001 by the National Institute for Health and Welfare (previously the National Public Health Institute). The data were collected according to two-stage stratified cluster sampling and were representative of the Finnish adult population aged ≥ 30 years [27]. In total, 6335 participants attended the general health and oral examinations. Panoramic radiograph was taken from 6114 of these participants. The subset of the data used in this study consists of 6331 participants from whom TMD signs were recorded (*N* = 6318) and/or TMJs could be assessed from panoramic radiographs (*N* = 5289) (Table 1).

**Table 1:** Number of observations (N) and characteristics of the study groups categorized according to inflammatory polyarthritis status.

	N (Total)	N (PTG)	Gender		p_x_	Removable dentures		p_x_	Smoking		p_x_	Age±SD	p_T/W_
			Males (%)	Females (%)		No (%)	Yes (%)		No (%)	Yes (%)			
No inflammatory polyarthitis	6146	5140	45.7	54.3		68.6	31.4		78.3	21.7		53 ± 15	
At risk of RA	47	35	31.9	68.1	0.059	30.4	69.6	<0.001	76.6	23.4	0.773	65 ± 16	<0.001^T^
Polyarthritis other than RA	44	35	54.5	45.5	0.239	62.8	37.2	0.415	84.1	15.9	0.355	55 ± 16	0.279^T^
Seronegative RA	58	50	19.0	81.0	<0.001	43.1	56.9	<0.001	89.7	10.3	0.037	63 ± 16	<0.001^T^
Seropositive RA	36	29	27.8	72.2	0.032	52.8	47.2	0.042	80.6	19.4	0.747	61 ± 12	<0.001^W^
**Total**	**6331**	**5289**	**45.3**	**54.7**		**67.9**	**32.1**		**78.5**	**21.5**		**53 ± 15**	

p_x_= *p*-values are based on chi-square tests for associations between inflammatory polyarthritis groups and gender, removable dentures or smoking. Other inflammatory polyarthritis groups were compared to the “no inflammatory polyarthritis” group.

p_T/W_: *p*-values are based on *t*-tests (^T^) or Welch’s test (^W^) for associations between age and inflammatory polyarthritis groups. Other inflammatory polyarthritis groups were compared to the “no inflammatory polyarthritis” group.

PTG: panoramic tomography of temporomandibular joint; RA: rheumatoid arthritis.

TMD examinations were conducted as part of standardized clinical oral examinations by trained dentist-nurse teams to 6318 participants [27]. TMD examinations included recording of maximum interincisal distance, auscultation of TMJ sounds (clicking, crepitation) and palpation of TMJs and two masticatory muscles (*m. temporalis anterio*r and *m. masseter superficialis*). Maximum interincisal distance was measured with a ruler without overbite. Mouth opening was considered restricted if maximum interincisal distance was <40 mm. Pain in TMJs during palpation was assessed by applying a force of about 0.5 kg over the immovable condyle. Palpation pain in masticatory muscles was assessed with a force of about 1 kg. Attempts were made to standardize the palpation force by exerting the forces on a measuring scale between the examinations. Pain on palpation was recorded if the subject reported pain when asked or showed a protective reflex. Although TMJ sounds and palpation pain were recorded separately for both sides during examinations, they were combined in the results and recorded as either present or absent. For the statistical analyses 5 dichotomous response variables of TMD signs were formed: reduced mouth opening, clicking, crepitation, pain in joints, pain in muscles. Also, information on removable dentures were obtained during clinical examinations. Various steps were taken to assure the quality of the examination procedures and the measurements prior and during the field examinations: e. g. all oral health teams received a two-week training and a dry run in real field circumstances. During the field stage the quality assurance included repeat and parallel measurements, spread evenly throughout the field stage of the survey. The quality assurance protocol is described in detail in [28].

Of the total 6114 digital panoramic radiographs (Planmega 2002 CC Proline) obtained from the subjects, 5289 could be used in this study. Panoramic radiographs were included in the study if the general quality of the radiograph was sufficient for analyses and one or both TMJs were fully visible. Radiographs were first interpreted by the dentist who made the clinical examination and later by three specialists in oral radiology. Kappa value (± 95% CL) for the findings in TMJs was 0.30 (± 0.7). Abnormal findings were recorded, and they were also categorized to radiological findings related to arthrosis or arthritis. Radiological findings related to arthrosis were set based on two of the following criteria: 1) flattening, unevenness or sclerosis of the condyle, 2) osteophytes in the condyle, or 3) erosion or sclerosis of the articular eminence. Radiological findings related to arthritis were set when erosion of the condyle or cyst-like formation in the condyle was found.

Inflammatory polyarthritis was recorded during the clinical examinations of musculoskeletal system by a specially trained physician [29]. The diagnoses made were based on medical history, symptoms and clinical findings of inflammatory poly-arthritis. Also, the level of rheumatoid factor (RF) was analyzed from fasting serum samples. Levels of anti-cyclic citrullinated peptides (aCCP) were analyzed from all RF-positive serum samples [29]. Subjects were categorized with regard to inflammatory polyarthritis according to following criteria: 1) no inflammatory polyarthritis according to clinical and serological findings (reference group); 2) ‘subjects at risk of RA’ having systemic autoimmunity associated with RA (aCCP confirmed seropositivity indicating possible subclinical disease (RF> 25 IU/ml and aCCP > 20 U/ml)) but no clinical findings; 3) inflammatory arthritis other than RA (including ankylosing spondylitis) according to clinical findings and seronegativity (RF< 25 IU/ml); 4) seronegative RA based on clinical findings and seronegativity; 5) aCCP confirmed sero-positive RA (clinical findings, RF > 25 IU/ml and aCCP > 20 U/ ml). Information on the smoking habit was also obtained, as polyarthritis is associated with smoking [30]. Smoking was inquired based on a questionnaire and was categorized to smoking daily, smoking occasionally, has quitted smoking and non-smoker.

### Statistical methods

Age, gender, smoking history and removable dentures were assumed to act as possible confounding factors based on prior knowledge of the subject. Chi-square tests were used to evaluate the statistical significance of associations of these factors with TMD signs and with inflammatory polyarthritis groups. Association of age with polyarthritis groups and with TMD signs were tested using independent samples *t*-test (when variances were equal according to Levene’s test) and Welch’s test (in the cases of unequal variances).

To evaluate the association of inflammatory polyarthritis groups with TMD signs and radiological findings chi square tests or Fisher’s exact tests (when assumptions of chi-square test were not met) were used. Separate logistic regressions were used to evaluate the associations of inflammatory poly-arthritis groups with each TMD signs while adjusting for the confounding factors. First order interaction terms between inflammatory polyarthritis group and covariates were tested for statistical significance, but they were excluded as none was statistically significant. Results are presented in terms of odds ratios (ORs) with 95% confidence limits (CL). The statistical analyses were conducted using IBM SPSS Statistics version 20 (IBM Corporation).

## Results

Characteristics (gender, dentures, smoking and age) of the inflammatory polyarthritis groups are presented in Table 1. Subjects with seronegative or seropositive RA were more often women, had more often dentures and were older than subjects without inflammatory polyarthritis. The subjects at risk of RA also had more often dentures and were older than subjects without polyarthritis. According to bivariate analyses, all confounding factors were associated with both inflammatory polyarthritis groups (explanatory variable, Table 1) and TMD signs (response variable, Table 2) in varying degrees.

**Table 2:** Associations of gender, removable dentures, smoking status and age with TMD signs and abnormal radiological findings.

	Restricted mouth opening (< 40 mm)			Clicking in TMJ			Crepitation in TMJ			Pain in TMJ			Pain in MM			Abnormal radiological finding		
	no	yes	*p*	no	yes	*p*	no	yes	*p*	no	yes	*p*	no	yes	*p*	no	yes	*p*
Gender																		
Males (%)	93.7	6.3	<0,001	87.1	12.9	<0.001	94.7	5.3	<0.001	97.7	2.3	<0,001	92.1	7.9	<0.001	92.7	7.3	<0,001
Females (%)	87.9	12.1		82.2	17.8		89.6	10.4		94.7	5.3		79.7	20.3		85.2	14.8	
Dentures																		
No (%)	93.7	6.3	<0,001	85.2	14.8	0.016	92.3	7.7	0.098	96.7	3.3.	<0,001	88.9	11.1	<0.001	88.9	11.1	0.097
Yes (%)	83.8	16.2		82.8	17.2		91.1	8.9		94.7	5.3		77.7	22.3		87.3	12.7	
Daily smoker																		
No (%)	89.9	10.1	0.002	84.1	15.9	0.15	91.5	8.5	0.016	95.7	4.3	0.002	84.3	15.7	<0.001	88.1	11.9	0.238
Yes (%)	92.8	7.2		85.7	14.3		93.5	6.5		97.5	2.5		89.1	10.9		89.4	10.6	
Age ± SD	52 ± 15	62 ± 15	<0,001^W^	53 ± 15	53 ± 15	0.268^T^	52 ± 15	58 ± 15	<0,001^T^	53 ± 15	57 ± 16	<0,001^W^	52 ± 14	60 ± 17	<0,001^W^	52 ± 14	55 ± 15	<0,001^T^

Differencies in frequency distributions were tested with Χ^2^-tests.

^T^: differencies in means were tested with *t*-test; ^W^: differencies in means were tested with Welch’s test.

TMJ: temporomandibular joint; MM: masticatory muscles (m. masseter and m. temporalis).

In the bivariate analyses, inflammatory polyarthritis groups were statistically significantly associated with abnormal radiological findings and all the measured TMD signs except TMJ clicking (Table 3). Of the subjects with inflammatory polyarthritis, ≥60% had at least 1 TMD sign (Table 3). In comparison, 38.0% of the subjects without polyarthritis had at least 1 TMD sign. When both TMD signs and radiological findings were considered, 68.8% of the subjects with seronegative RA and 66.7% with seropositive RA had at least one finding. Abnormal radiological finding was the most frequent finding in subjects with seronegative (36.0%) and seropositive RA (32.1%), whereas TMJ clicking was the most frequent finding in subjects without polyarthritis (15.5%). Radiological findings related to arthritis were infrequent and mostly found within subjects with seronegative or seropositive RA. Radiological changes related to arthrosis accounted for most of the abnormal radiological findings and were frequently found in sero-negative (32.0%) and seropositive (32.1%) RA subjects.

**Table 3:** Frequencies [*n*(%)] of clinical signs of temporomandibular disorders (TMD) and radiological findings in temporomandibular joint (TMJ) in relation to inflammatory polyarthritis.

	TMD signs										Panoramic radiograph of TMJ				≥1 TMD signs (%)	≥1 TMD and PTG findings (%)
	Restricted opening		Clicking		Crepitation		TMJ pain		Pain in MM		Abnormal finding		Arthritis	Arthrosis		
	*n*	(%)	*n*	(%)	*N*	(%)	*n*	(%)	*n*	(%)	*n*	(%)	%	%		
No inflammatory polyarthritis	552	(9.1)	950	(15.5)	480	(7.8)	233	(3.8)	880	(14.3)	579	(11.3)	0.5	10.4	38.0	42.5
At risk of RA	9	(19.6)	8	(17.4)	5	(10.9)	2	(4.3)	12	(26.1)	4	(11.4)	0.0	11.4	50.0	50.0
Polyarthritis other than RA	10	(23.3)	9	(20.9)	3	(7.0)	4	(9.3)	8	(18.6)	2	(5.7)	0.0	5.7	60.5	58.8
Seronegative RA	14	(25.0)	7	(12.1)	12	(20.7)	6	(10.3)	17	(29.3)	18	(36.0)	2.0	32.0	64.3	68.8
Seropositive RA	8	(22.9)	9	(25.0)	10	(27.8)	4	(11.1)	10	(27.8)	9	(32.1)	3.6	32.1	60.0	66.7
*P*	<0.001*		0.400		<0.001*		0.006*		<0.001*		<0.001*		0.155*	<0.001*	<0.001	<0.001*

p-values are based on chi-square tests or Fisher’s exact tests (*) for associations between inflammatory polyarthritis groups and TMD signs.

Restricted opening: maximal interincisal opening <40 mm; MM: masticatory muscles (*m. temporalis* and *m. masseter*); ≥1 TMD signs (%): percentage of subjects having at least one of 5 TMD signs; ≥1 TMD and PTG findings (%): percentage of subjects having at least one TMD sign or abnormal finding in panoramic radio-graph; RA: rheumatoid arthritis.

Based on the logistic regressions, seronegative RA associated statistically significantly with restricted mouth opening, TMJ crepitation and abnormal radiological findings (Table 4). Seropositive RA also associated significantly with TMJ crepitation and abnormal radiological findings. In general, subjects with RA had over 3 times the risk of abnormal radiological findings compared to subjects without inflammatory polyarthritis. Polyarthritis other than RA was associated with restricted mouth opening. Systemic autoimmunity related to serum RF and aCCP levels (‘at risk of RA’) was not associated with the clinical or radiological TMD findings.

**Table 4:** Logistic regressions of association of inflammatory polyarthritis with clinical and radiological TMD signs.

	Restricted mouth opening			Clicking in TMJ			Crepitation in TMJ			Pain in TMJ			Pain in masticatory muscles			Abnormal radiological findings		
	OR	CL^L^	CL^U^	OR	CL^L^	CL^U^	OR	CL^L^	CL^U^	OR	CL^L^	CL^U^	OR	CL^L^	CL^U^	OR	CL^L^	CL^U^
Inflammatory																		
arthritis status																		
(ref. “none”)																		
At risk of RA	1.29	0.60	2.76	1.01	0.47	2.19	1.08	0.42	2.78	0.87	0.21	3.64	1.20	0.60	2.39	0.85	0.29	2.46
Other than RA	3.13	1.48	6.63	1.49	0.71	3.12	0.89	0.27	2.91	2.70	0.95	7.70	1.43	0.64	3.19	0.51	0.12	2.13
Seronegative RA	1.97	1.04	3.72	0.67	0.30	1.48	2.23	1.16	4.29	2.08	0.88	4.95	1.44	0.80	2.60	3.41	1.88	6.19
Seropositive RA	2.21	0.98	4.98	1.69	0.79	3.63	3.69	1.75	7.78	2.58	0.90	7.44	1.67	0.79	3.53	3.14	1.40	7.05
Age (years)	1.04	1.03	1.04	1.00	0.99	1.00	1.03	1.02	1.03	1.00	0.99	1.01	1.03	1.02	1.04	1.02	1.01	1.02
Female gender (ref. male)	1.80	1.47	2.20	1.42	1.22	1.64	1.95	1.58	2.41	2.11	1.56	2.86	2.64	2.23	3.14	2.10	1.72	2.55
Dentures (ref. “none”)	1.45	1.17	1.79	1.21	1.02	1.45	0.67	0.53	0.84	1.40	1.02	1.94	1.29	1.08	1.54	0.80	0.64	1.01
Smoking (reference “no”)																		
daily	1.10	0.86	1.42	0.95	0.79	1.14	1.05	0.81	1.36	0.66	0.44	0.97	0.99	0.80	1.21	1.08	0.86	1.36
occasionally	0.87	0.48	1.57	1.44	1.04	2.00	0.93	0.54	1.60	0.66	0.28	1.52	0.93	0.59	1.45	0.78	0.46	1.30
has quitted	1.00	0.79	1.26	0.92	0.76	1.11	0.97	0.76	1.25	0.98	0.70	1.38	0.90	0.74	1.10	0.88	0.69	1.12

TMD: temporomandibular disorders; Restricted mouth opening: maximum interincisal distance <40 mm); TMJ: temporomandibular joint; masticatory muscles: *m. temporalis* and *m. masseter*; RA: rheumatoid arthritis; OR: odds ratio; CL^L^: lower confidence limit (95%); CL^U^: upper confidence limit (95%).

## Discussion

Our study on the Finnish adult population indicates that RA is associated with both radiological TMJ findings and clinical signs of TMD. *Ca*. two-thirds of subjects with RA had at least one clinical or radiological finding of TMD. The most prevalent finding among subjects with RA was abnormal findings in panoramic radiographs (in over 30% of the studied subjects). In comparison, for subjects without inflammatory poly-arthritis clicking of TMJ was the most prevalent finding. This study also indicates that although subjects with inflammatory polyarthritis frequently have also other signs of TMD, these findings commonly are related to other factors such as gender, age and denture status. Indeed, both RA and TMD are more frequent among women and prevalence increases with age [7,8,31]. Therefore, controlling several background factors is important when studying the relationship between inflammatory polyarthritis and TMD.

According to our results, RA increased the risk of abnormal radiological findings and crepitation of the TMJ. Restricted mouth opening was also associated with seronegative RA. In fact, restricted opening and TMJ crepitation have been found to correlate well with panoramic radiography and MRI findings of disc abnormalities and degeneration of TMJ surfaces related to inflammatory polyarthritis [13,14]. Our results therefore support the previous findings, that clinical examination is a suitable method for screening patients and especially limited mouth opening and TMJ crepitation in subjects with polyarthritis should lead to further examination and treatment of pathological changes in the TMJ [13,14]. Findings in panoramic radiograph related to arthritis were infrequent but almost exclusively found in subjects with RA in the present study. Also, findings of arthrosis were more frequent among subjects with RA than among subjects with other inflammatory polyarthritis or in general population. Cone beam computed tomography CBCT is a sensitive method to find changes in TMJ related to the polyarthritis [32,33], and Cordeiro et al. [33] reported all studied RA subjects having either clinical or radiological findings indicative of degenerative changes in the TMJ.

There exists only very limited knowledge on the frequency of TMD in subjects having increased risk of developing RA due to the positive levels of related serum autoantibodies, namely RF and aCCP [20,26]. In this study, increased risk of RA was not associated with the clinical or radiological TMD findings. Also, Kim et al. [34] did not find more frequent TMD signs or symptoms in subjects with elevated RF in general. However, they reported that males with elevated serum antibodies (RF and ANA in their study) were associated to restricted mouth opening and pain on neck muscle palpation. In contrast, Kroese et al. [26] reported higher prevalence of painful TMD in individuals at risk of RA compared to the healthy controls, whereas in non-painful TMD similar difference was not found. These contrasting findings could relate to differences in study design: in addition to autoantibodies, subjects at the ‘risk of RA’ group by Kroese et al. [26] also had arthralgia diagnosed by a physician at the rheumatology clinic. Thus, the pathological process of developing RA may be more pronounced in the subjects of Kroese et al. [26]. In subjects with diagnosed RA, previous studies report that elevated levels of RF and aCCP are related to more frequent clinical signs and symptoms of TMD compared to controls [20,32,35].

Restricted mouth opening was associated with inflammatory polyarthritis in this study, although the for seropositive RA group this association was not statistically significant. Several previous studies also report reduced maximal mouth opening in RA subjects [13–15,18,36], as well as in subjects with other inflammatory polyarthritis [13,37]. Yet, Yamakawa et al. [38] reported no significant reduction in the mouth opening between RA subjects and the control group. The pathological processes behind restricted mouth opening can relate to changes in TMJ (such as degenerative changes in the articular fibrocartilage, displacement of the articular disc and fibrotic adhesions i the joint) or to extra-articular causes (most commonly to the muscular pain)[39].

The frequency of TMJ crepitation among subjects with RA (21% in seronegative RA and 28% seropositive RA) was roughly the same order of magnitude as reported in many studies previously (14– 36%; [16,18,38,40]. In contrast, Helenius et al. [13,14] reported clearly higher frequencies (75%) among subjects with RA. Crepitation of TMJ often indicates structural damage by degenerative joint disease [14,41]. In our study, subjects with RA were over two times more likely to have crepitation compared to the general population (seronegative RA: odds ratio 2.2 and seropositive RA: odds ratio 3.7).

The prevalence of TMD in the adult Finnish population according to the Health 2000 survey (data of which was used also in this study) has been reported by Rutkiewicz et al. [7]. The proportion of subjects having at least one TMD sign was 38% [7]. This is comparable to 35% reported by Qvintus et al. [8] based on the Health 2011 survey, which also is representative of the Finnish adult population. Compared to these figures representing the population in general, proportion of subjects with inflammatory polyarthritis having at least one TMD sign was higher: inflammatory polyarthritis other than RA 61%, seronegative RA 64% and seropositive RA 60%, indicating that even two-thirds of subjects with polyarthritis have clinical signs of TMD. Results of most previous studies indicate that more than half of patients with RA exhibit clinically evident TMJ involvement [13–16,18,20,42]. Yet, large range of frequencies have been reported due to differences in study design and diagnostic criteria (for example Lin et al. [19]). Also, Jalal et al. [35] reported an increasing frequency of TMD signs with regard to the time of diagnosing the RA: 15% of subjects with RA diagnosed within 5 years had TMD signs compared to 40% of subjects with RA diagnosed within 6–10 years. This implies that majority of subjects with RA have some signs of TMD and those who could benefit from further TMD examination and management should be identified and directed to a suitable dental clinic.

Utilizing data from a large and representative sample of Finnish adults was one of the main strengths of this study. Considerable efforts were made to assure the quality of the examination procedures and the measurements. However, there are also certain limitations considering the dataset. The Health 2000 Survey did not consider symptoms of TMD reported by the subjects. In general, symptoms of TMD can be more infrequent than signs [8] and, also among subjects with polyarthritis many of the signs can remain asymptomatic [15,43]. It should be noted that setting a TMD clinical sub-diagnosis is based both on TMD signs and symptoms according to the DC/TMD criteria [1]. These criteria were not published at the time of the data collection and therefore they were not used in the present study. In TMD diagnostics, different time frames for TMD symptom report are used, f. ex 30 days in the DC/TMD diagnostics [1]. Clinical examination of masticatory system was part of wider health examination lasting for 4 h per subject. Due to practical time limitations, the present study only assessed the current TMD findings, which did not allow deriving any diagnoses.

In conclusion, the results of this study on the Finnish adult population support previous findings that clinical and radiological findings of TMD are more prevalent among subjects with inflammatory polyarthritis than among the population in general. Inflammatory polyarthritis increased the risk of restricted mouth opening and crepitation of TMJ as well as abnormal radiological findings in panoramic tomography. This study adds to the few previous studies reporting on the TMD at subjects at risk of developing inflammatory polyarthritis based on the increased levels of RF and aCCP antibodies. In the present study, subjects ‘at risk’ did not differ from the general population in TMD signs or radiological findings. The study findings indicate that TMD is commonly manifested in subjects with RA. In the case of abnormal radiological TMJ findings and clinical signs, suspect of RA should be taken into account, and the patient should be referred and treated in multidisciplinary collaboration when needed. On the other hand, RA patients should be examined and treated by dentists for detecting the possible TMD findings and their consequences, such as occlusal changes.
